# Itch and autophagy-mediated NF-κB activation contributes to inhibition of cathepsin D-induced sensitizing effect on anticancer drugs

**DOI:** 10.1038/s41419-022-05011-4

**Published:** 2022-06-17

**Authors:** Seung Un Seo, Seon Min Woo, Kyoung-jin Min, Taeg Kyu Kwon

**Affiliations:** 1 Department of Immunology, School of Medicine, Keimyung University, Daegu, 42601 South Korea; 2 New Drug Development Center, Daegu-Gyeongbuk Medical Innovation Foundation (DGMIF), Daegu, 41061 South Korea; 3 Center for Forensic Pharmaceutical Science, Keimyung University, Daegu, 42601 South Korea

**Keywords:** Chemotherapy, Ubiquitylation, Autophagy

## Abstract

Inhibition of cathepsin D (Cat D) sensitizes cancer cells to anticancer drugs via RNF183-mediated downregulation of Bcl-xL expression. Although NF-κB activation is involved in the upregulation of RNF183 expression, the molecular mechanism of NF-κB activation by Cat D inhibition is unknown. We conducted this study to investigate the molecular mechanism underlying Cat D-mediated NF-κB activation. Interestingly, Cat D inhibition-induced IκB degradation in an autophagy-dependent manner. Knockdown of autophagy-related genes (*ATG7* and *Beclin1*) and lysosome inhibitors (chloroquine and bafilomycin A1) blocked IκB degradation via Cat D inhibition. Itch induced K63-linked ubiquitination of IκB and then modulated the protein stability of IκB by Cat D inhibition. Inhibition of Cat D-mediated Itch activation was modulated by the JNK signaling pathway, and phosphorylated Itch could bind to IκB, resulting in polyubiquitination of IκB. Additionally, inhibition of Cat D increased autophagy flux via activation of the LKB1-AMPK-ULK1 pathway. Therefore, our results suggested that Cat D inhibition activated NF-κB signaling via degradation of autophagy-dependent IκB, which is associated with the upregulation of RNF183, an E3 ligase of Bcl-xL. Cat D inhibition enhances TRAIL-induced apoptosis through Bcl-xL degradation via upregulation of RNF183.

## Introduction

Cathepsin D (Cat D) is highly expressed in several cancers [1–7], and it is involved in tumor-promoting effects, such as an increased invasion/migration [8–10] and resistance to anticancer drugs [11]. Inhibition of Cat D induces H_2_O_2_-induced apoptosis in HeLa cells [12] and sensitizes neuroblastoma cells to doxorubicin [11]. Particularly, the pro-tumoral effects of Cat D are well known in breast cancer. Cat D is a marker for poor prognosis in breast cancer [13, 14], and Cat D deficiency in the mammary epithelium transiently delays breast cancer progression by inhibiting mTORC1 signaling [15]. Cat D inhibition sensitizes cancer cells to anticancer drugs via RNF183-mediated Bcl-xL degradation [16]. The upregulation of RNF183, which is a key E3 ligase for Bcl-xL degradation, is mainly regulated by activation of the NF-κB signaling pathway [16]. However, the molecular mechanisms underlying NF-κB activation with Cat D inhibition remain unclear.

NF-κB is an important transcription factor involved in multiple cellular functions [13–17]. In the resting state, the IκB-NF-κB complex exists in an inactive form in the cytoplasm. Several stimuli, such as inflammatory cytokines, induce IκB phosphorylation, resulting in the degradation of IκB [17]. The released NF-κB translocates from the cytosol to the nucleus, binds to the target gene promoter, and regulates gene expression. Therefore, degradation of IκB is essential for the activation of NF-κB signaling, and it is mainly mediated via the ubiquitin-proteasome pathway [18]. Moreover, β-TrCP is a major E3 ligase for IκB degradation [19–21]. When IκB is phosphorylated at S32 and S36 through stimuli, β-TrCP induces K48-linked polyubiquitination at K21 and K22 [22].

In this study, we investigated the molecular mechanism of IκB degradation through Cat D inhibition and identified a novel mechanism of autophagy-lysosome-dependent IκB degradation. NF-κB activation contributed to the upregulation of RNF183, and RNF183-mediated destabilization of Bcl-xL played a critical role in the sensitizing effect of Cat D on anticancer drugs.

## Materials and methods

### Cell culture and materials

Human cancer cell lines Caki, HT29, DU145 and A549 were obtained from the American Type Culture Collection (Manassas, VA, USA). All cells were cultured in Dulbecco’s modified Eagle’s medium containing 10% fetal bovine serum (Welgene, Gyeongsan, Korea), 1% penicillin–streptomycin, and 100 μg/mL gentamycin (Thermo Fisher Scientific, Waltham, MA, USA). Details of the reagents, antibodies, siRNAs, and plasmids used are provided in Supplementary Table 1.

### Generation of Cat D knockout (KO) cells

Two CRISPR sgRNAs, oligomer1, 5-CAC CGA TGG GCC CCT CGG TCA CGG C-3′ and oligomer2, 5-AAA CGC CGT GAC CGA GGG GCC GAT C-3′, were designed using CRISPR. The cell lines were established by transfecting sgRNAs into Caki cells using the Lipofactor-pMAX reagent (AptaBio, Yongin, Korea). Cells were selected on 0.5 μg/mL puromycin, and Cat D KO efficiency was analyzed using western blot analysis.

### Western blot analysis

Cells were collected and lysed in RIPA lysis buffer, and the lysates were centrifuged at 13,000 × *g* and 4 °C for 15 min [23]. The supernatants were collected and boiled in 5× sample buffer at 95 °C for 5 min. Proteins were separated using SDS-PAGE and transferred onto nitrocellulose membranes (GE Healthcare Life Sciences, Pittsburgh, PA, USA). The protein bands were detected using an enhanced chemiluminescence reagent kit (EMD Millipore, Darmstadt, Germany).

### Ubiquitination assay

Ubiquitination assay was performed as previously described [24]. Cells were co-transfected with an HA-tagged ubiquitin (HA-Ub) plasmid, and they were treated with MG132 for 12 h. Immunoprecipitation was performed using anti-IκB, and ubiquitination of endogenous IκB was assessed using an HRP-conjugated anti-Ub antibody under denaturing conditions.

### Immunoprecipitation assay

Cells were collected, washed with PBS, lysed with RIPA lysis buffer containing 10 mM N-ethylmaleimide (EMD Millipore) and 1 mM PMSF, and sonicated on ice for protein extraction. After sonication, the cell lysates were centrifuged at 13,000 × *g* and 4 °C for 15 min. The supernatants were incubated with 1 μg anti-IκB antibody at 4 °C overnight, and they were then attached to 20 μL of Protein G agarose beads by mixing on a rotator at 4 °C for 2 h. Cell lysates were washed with RIPA lysis buffer containing 10 mM N-ethylmaleimide and 1 mM PMSF (Sigma-Aldrich, St. Louis, MO, USA), and they were boiled in 2× sample buffer for 10 min. The protein–protein interactions were verified via western blot analysis.

### Transfection and luciferase assay

The cells were transfected with siRNA using Lipofectamine RNAiMAX (Thermo Fisher Scientific), and they were transiently transfected with the promoter plasmid using Lipofectamine^TM^ 2000 (Thermo Fisher Scientific). After treatment, the cells were collected and lysed in a lysis buffer (25 mM Tris-phosphate, pH 7.8, 2 mM EDTA, 10% glycerol, and Triton X-100). The supernatant was analyzed using a dual-luciferase reporter reagent (Promega, Madison, WI, USA) according to manufacturer’s recommendations.

### mRFP-EGFP-LC3 puncta

The cells were transiently transfected with mRFP-EGFP-LC3 using Lipofectamine^TM^ 2000. Following drug treatment, the cells were mounted using ProLong Gold (Thermo Fisher Scientific). Fluorescence signals were captured using a confocal microscope.

### Nuclear translocation assay

The treated cells were fixed with 4% paraformaldehyde on glass slides at room temperature for 30 min, washed with PBS, and permeabilized with 1% Triton X-100 at room temperature for 1 min. After washing with PBS, the slides were stained with an anti-TFEB antibody at 4 °C overnight. Subsequently, the slides were incubated with an FITC-conjugated secondary antibody for 1 h, washed with PBS, and mounted on coverslips with ProLong™ Gold Antifade Mountant with DAPI (Thermo Fisher Scientific). Nrf2 localization was determined using an LSM 510 multiphoton confocal microscope (Carl Zeiss, Jena, Germany).

### FACS analysis for detection of cell death

Cells were harvested and resuspended in 100 μL PBS, and 200 μL of 95% ethanol were added at 4 °C for 1 h. Following this, cells were resuspended in 1.12 % sodium citrate buffer (pH 8.4) containing 12.5 μg of RNase at 37 °C for 30 min, after which 50 μg/mL of propidium iodide solution were added. The apoptotic population was assessed using a BD Accuri^TM^ C6 flow cytometer (BD Biosciences, San Jose, CA, USA).

### Animals

Male BALB/c-nude mice were purchased from the Central Lab Animal, Inc. (Seoul, Korea). For acclimatization, they were kept at 25 ± 2 °C under a relative humidity of 55 ± 5% and 12 h light/dark cycle for one week. The study protocol was approved by the Institutional Review Board (KM-2015-03R2) of Keimyung University ethics committee.

### In vivo xenograft model

Experiments were performed as previously described [25]. Male NOD/SCID mice were subcutaneously injected with 5 × 10^6^ Caki cells into the flack of each mice. After 3 weeks, mice were assigned to the following experimental groups: vehicle, 3 mg/kg Pep A (20% DMSO + PBS), 3 mg/kg GST-TRAIL, a combination of Pep A and GST-TRAIL. Mice were intraperitoneally injected three times a week for 16 days, and the modulation of protein expression was identified using the same sample used previously [16]. All groups did not change mortality in mice until the end of the study.

### Patient specimens

A total of 40 patients diagnosed with RCC were included in this retrospective study. RCC tissues were collected from patients undergoing surgery in Keimyung University Dongsan Medical Center (Daegu, Korea) and provided by the Biobank of Keimyung University Dongsan Hospital Biobank (IRB-2019-11-040). We identified the related proteins using samples from patients used in previous experiments [16].

### Statistical analysis

Data were analyzed using one-way ANOVA and post-hoc comparisons (Student–Newman–Keuls) using the Statistical Package for Social Sciences software (version 22.0; SPSS Inc. Chicago, IL, USA).

## Results

### Inhibition of Cat D activates the NF-κB signaling pathway via autophagy-dependent IκB degradation

**Fig. 1:**
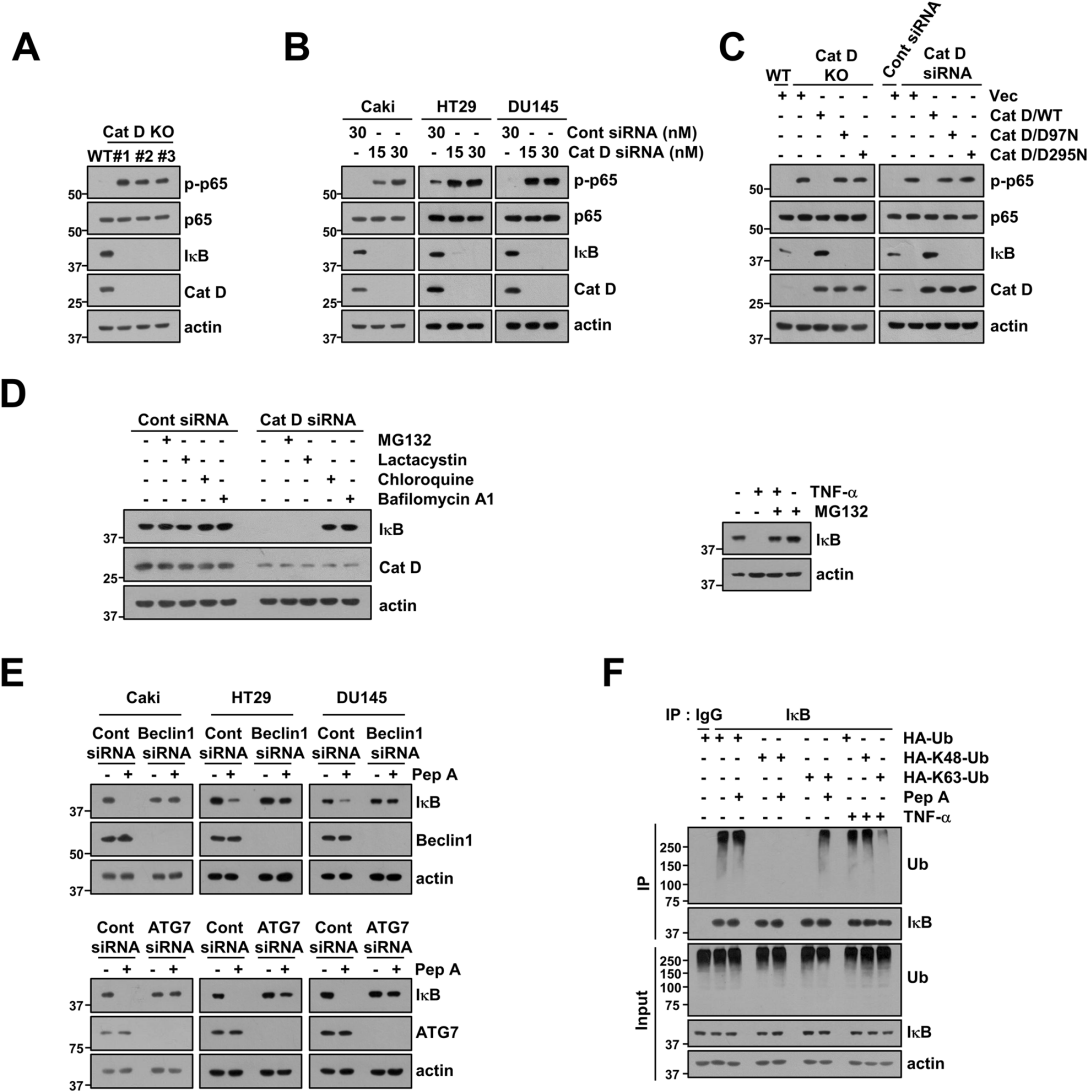
Inhibition of cathepsin D (Cat D) induces IκB degradation via the autophagy-lysosome pathway. **A**, **B** Examination of protein expression in Caki/Cat D WT and KO cells (**A**) or Cont and Cat D siRNA-transfected cells (**B**). **C** Caki/Cat D KO cells and Cat D siRNA-transfected cells were transfected with vector, Cat D WT, or Cat D mutants (D97N and D295N), and protein expression was determined. **D** Caki cells were transiently transfected with Cont siRNA or Cat D siRNA, and they were treated with 0.5 μM MG132, 2.5 μM lactacystin, 10 μM chloroquine, and 5 nM bafilomycin A1 for 24 h (left panel). Caki cells were treated with 0.5 μM MG132 for 30 min, and 20 ng/mL TNF-α was then added for 24 h (right panel). **E** Cancer cells (Caki, HT29 and DU145) were transiently transfected with Cont siRNA, Beclin1 siRNA, and ATG7 siRNA, and they were further treated with 2 μM Pep A for 24 h. **F** Caki cells were transiently transfected with HA-Ub, HA-K48-Ub, and HA-K63-Ub, and they were pretreated with 0.5 μM MG132 and 5 nM bafilomycin A1. Thereafter, they were treated with 2 μM Pep A or 20 ng/mL TNF-α for 24 h. The ubiquitination of endogenous IκB was analyzed via the DUB assay. Protein expression was measured via western blotting (**A**–**F**).

Inhibition of Cat D sensitizes cancer cells to anticancer drugs via downregulation of Bcl-xL while upregulation of RNF183 plays a critical role in Bcl-xL degradation [16]. Since inhibition of Cat D increased RNF183 mRNA expression by activating the NF-κB signaling pathway, we examined how inhibition of Cat D activates NF-κB signaling. As shown in Fig. 1A, B, Cat D knockdown or knockout (KD or KO) increased the phosphorylation of p65 and degradation of IκB proteins in all tested cancer cell lines. Moreover, the catalytically inactive mutants of Cat D, D97N and D295N, also activated NF-κB signaling (Fig. 1C). NF-κB activation is commonly induced by IκB degradation in a proteasome-dependent manner. However, proteasome inhibitors MG132 and lactacystin had no effect on the degradation of IκB in Cat D KD cells while MG132 blocked the tumor necrosis factor-α (TNF-α)-induced degradation of IκB (Fig. 1D). Therefore, we investigated whether the autophagy-lysosome pathway, another important proteolytic pathway, is involved in IκB degradation. Lysosome functional inhibitors chloroquine and bafilomycin A1 significantly reversed IκB degradation in Cat D KD cells (Fig. 1D). Furthermore, KD of autophagy-related genes *Beclin 1* and *ATG7* blocked the Cat D inhibitor pepstatin A (Pep A)-induced IκB degradation (Fig. 1E). K48-polyubiquitin chains mainly target proteins for proteasomal degradation, whereas K63-polyubiquitin chains modulate protein expression through proteasome-independent mechanisms such as autophagy-dependent degradation [26, 27]. Therefore, the ubiquitination chains of TNF-α and Pep A were compared. As anticipated, TNF-α boosted K48-polyubiquitin chains whereas Pep A boosted K63-polyubiquitin chains in the IκB protein (Fig. 1F). Therefore, our data suggested that inhibition of Cat D activates NF-κB signaling through autophagy-lysosome-dependent degradation of IκB.

### Itch is a E3 ligase for autophagy-lysosome degradation of IκB through Cat D inhibition

**Fig. 2:**
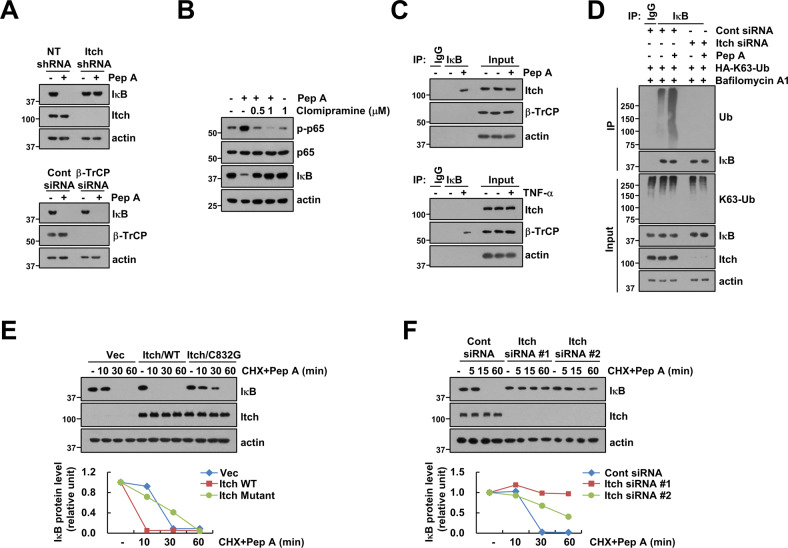
Itch is a E3 ligase for degradation of IκB through Cat D inhibition. **A** Caki cells were transduced with lentivirus containing either shRNA targeting Itch or a non-target sequence; alternatively, they were transiently transfected with Cont siRNA or β-TrCP siRNA and treated with 2 μM Pep A for 24 h. **B** Caki cells were pretreated with 0.5–1 μM clomipramine for 30 min, and they were then treated with 2 μM Pep A for 24 h. **C** Binding of IκB and Itch or β-TrCP was analyzed via the IP assay. **D** Caki cells were transiently co-transfected with Cont siRNA or Itch siRNA and HA-K63-Ub, and they were pretreated with 5 nM bafilomycin A1 for 30 min. They were then treated with 2 μM Pep A for 24 h. The ubiquitination of endogenous IκB was analyzed via the DUB assay. **E**, **F** Caki cells were transiently transfected with Itch WT and mutant (**E**) or Cont siRNA and two different Itch siRNA (**F**), and they were then treated with 20 μg/mL CHX and 2 μM Pep A for the indicated time points. Band intensity of Bcl-xL was analyzed using ImageJ. Protein expression was measured via western blotting (**A**–**F**).

Degradation of IκB by TNF-α is associated with β-TrCP-mediated ubiquitination [19]. However, the KD of β-TrCP did not alter the degradation of IκB through Cat D inhibition (Fig. 2A). Since inhibition of Cat D increased K63-linked ubiquitin chains of IκB (Fig. 1F), we investigated whether ubiquitination of IκB is induced by Itch E3 ligase, an E3 ligase that induces the formation of a K63-linked ubiquitin chain [27]. Itch KD or pharmacologic inhibitor (clomipramine) completely blocked IκB degradation and/or p65 phosphorylation following Pep A treatment (Fig. 2A, B). To confirm IκB degradation by Itch E3 ligase, we identified the interaction between Itch and IκB. Cat D inhibition increased IκB-Itch binding while TNF-α induced IκB-β-TrCP binding (Fig. 2C). Further, an increase in the K63-linked ubiquitin chain of IκB via Cat D inhibition was blocked by Itch KD (Fig. 2D). When de novo protein synthesis was blocked by cycloheximide treatment, overexpression of Itch dramatically decreased IκB protein stability; however, the catalytically inactive mutant Itch (C832G) inhibited IκB degradation in Pep A-treated cells (Fig. 2E). In contrast, KD of Itch increased IκB protein stability following Pep A treatment as compared to that of control siRNA (Fig. 2F). These data suggested that Itch is a critical E3 ligase that modulates Cat D inhibition-mediated IκB stability.

### Inhibition of Cat D increases activation of Itch via JNK-mediated phosphorylation of Itch at T222

**Fig. 3:**
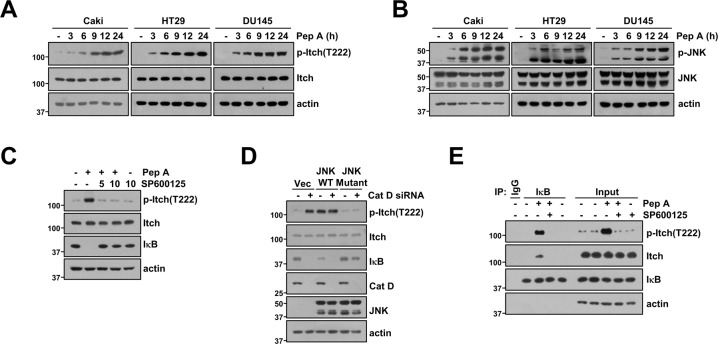
JNK activation is involved in Itch-mediated IκB degradation via Cat D inhibition. **A**, **B** Cancer cells (Caki, HT29, and DU145) were treated with 2 μM Pep A for the indicated time points. **C** Caki cells were pretreated with 10 μM SP600125 for 30 min, and they were then treated with 2 μM Pep A for 24 h. **D** Caki cells were transiently co-transfected with Cont siRNA or Cat D siRNA and JNK WT or JNK mutant for 24 h. **E** Caki cells were pretreated with 10 μM SP600125 for 30 min, and they were then treated with 2 μM Pep A. Binding of IκB and Itch or phospho-Itch was analyzed using the IP assay. Protein expression was measured via western blotting (**A**–**E**).

It has been reported that activation of Itch is regulated by JNK-dependent phosphorylation [28]. Therefore, we investigated whether inhibition of Cat D modulates phosphorylation of Itch. As shown in Fig. 3A and B, the extent of Itch phosphorylation at T222 significantly increased following Pep A treatment, which gradually enhanced JNK phosphorylation in all tested cancer cell lines. Furthermore, phosphorylation of Itch and degradation of IκB were completely blocked by a JNK-specific inhibitor (SP600125), and overexpression of kinase-inactive mutant JNK (JNK mutant) diminished Itch phosphorylation and IκB degradation by Cat D KD (Fig. 3C, D). Phosphorylation of Itch induced conformational changes related to E3 ligase activity. Therefore, we investigated whether phosphorylation of Itch plays a critical role in Itch-IκB binding. The JNK inhibitor diminished the interaction between Itch and IκB via inhibition of Itch phosphorylation (Fig. 3E). Therefore, our data suggested that JNK activation through Cat D inhibition plays a critical role in IκB degradation via phosphorylation of Itch at T222.

### Inhibition of Cat D increases autophagy flux

**Fig. 4:**
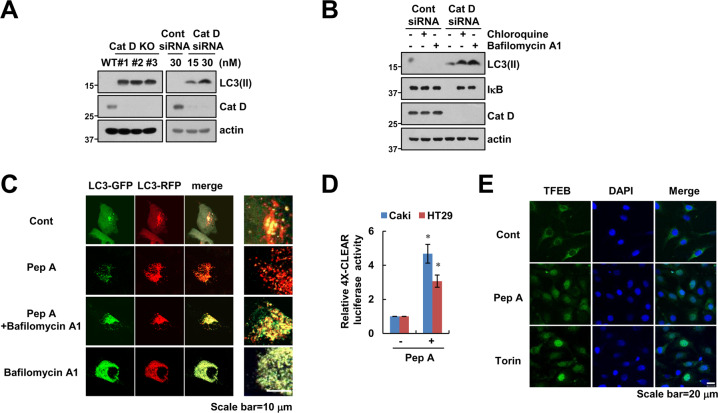
Inhibition of Cat D increases autophagy flux. **A** Caki KD and KO cells were used. **B** Caki cells were transiently transfected with Cont siRNA or Cat D siRNA, and they were treated with 10 μM chloroquine and 5 nM bafilomycin A1 for 24 h. **C** Caki cells were transiently transfected with mRFP-EGFP-LC3, and they were pretreated with 5 nM bafilomycin A1, following which they were treated with 2 μM Pep A for 18 h. mRFP-EGFP-LC3 puncta were observed via confocal microscopy. Scale bar = 10 μm. **D** Caki and HT29 cells were transiently transfected with a plasmid harboring the luciferase gene under the control of 4X-CLEAR plasmid, treated with 2 μM Pep A for 24 h, and then analyzed for luciferase activity. **E** Caki cells were treated with 2 μM Pep A for 6 h (positive control: 0.5 μM Torin). Translocation of TFEB was observed via confocal microscopy. Scale bar = 20 μm. Protein expression was measured via western blotting (**A**, **B**). Values in the graph (**D**) represent the mean ± SD of three independent experiments. *p < 0.01 compared to the control.

IκB degradation was mediated by the autophagy-lysosome-dependent pathway (Fig. 1D, E). Therefore, to evaluate the role of Cat D in autophagic flux [29], lipidated LC3 (LC3 II) in Cat D KO/KD cells was examined. LC3 II levels were higher in Cat D KO/KD cells than those in control cells (Fig. 4A), and lysosome inhibitors increased the levels of LC3 II (Fig. 4B). To further confirm this, cells were transfected with the mRFP-GFP-LC3 plasmid construct, which measures autophagic flux [30, 31]. Since the fusion of autophagosomes with lysosomes resulted in quenching of the GFP signal, autophagosomes (GFP+/RFP+) and autophagolysosomes (GFP−/RFP+) can be distinguished by the ratio of GFP and RFP signals. Pep A increased the number of autophagolysosomes (GFP−/RFP+), and the lysosome inhibitor bafilomycin A1 increased the number of autophagosomes (GFP+/RFP+) following Pep A treatment (Fig. 4C). Transcription factor EB (TFEB) is a master transcription factor involved in the induction of autophagy- and lysosomal biogenesis-related genes through binding to the coordinated lysosomal expression and regulation motif [32]. Pep A significantly increased the activity of TFEB (Fig. 4D) and induced nuclear translocation of TFEB (Fig. 4E). Therefore, our data suggested that inhibition of Cat D increases autophagic flux.

### LKB1-AMPK-ULK1 signal axis is involved in an increase of autophagy flux via Cat D inhibition

**Fig. 5:**
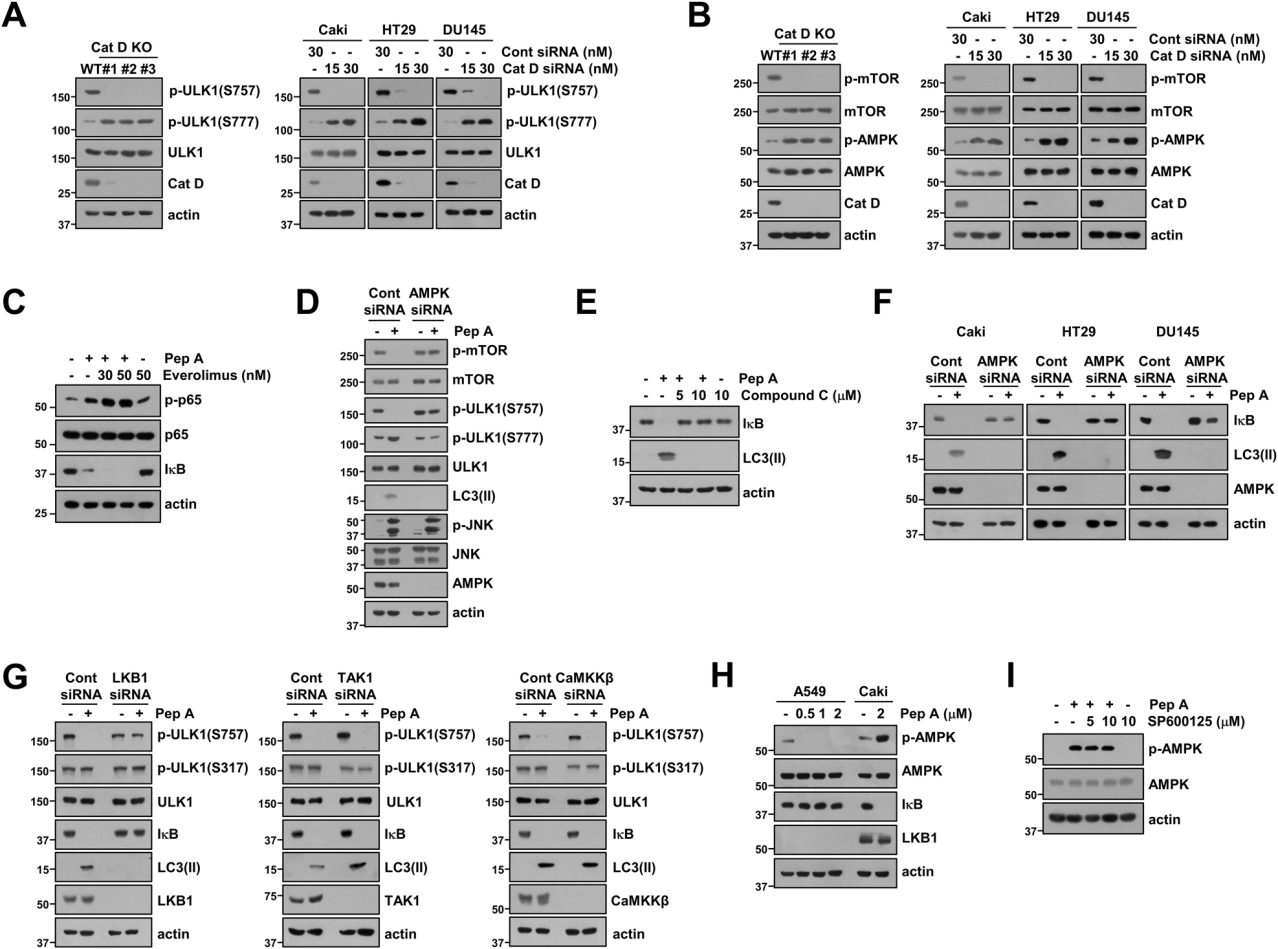
LKB1/AMPK/ULK1 signaling pathway is involved in Pep A-induced autophagy flux. **A**, **B** Examination of protein expression in Caki/Cat D WT and KO cells or Cont and Cat D siRNA-transfected cancer cells. **C** Caki cells were pretreated with the indicated concentrations of Everolimus, and 2 μM Pep A was then added for 24 h. **D** Caki cells were transiently transfected with Cont siRNA or AMPK siRNA, and they were treated with 2 μM Pep A for 24 h. **E** Caki cells were pretreated with the indicated concentrations of Compound C, and 2 μM Pep A was then added for 24 h. **F** Cancer cells (Caki, HT29, and DU145) cells were transiently transfected with Cont siRNA or AMPK siRNA, and they were then treated with 2 μM Pep A for 24 h. **G** Caki cells were transiently transfected with Cont siRNA, LKB1 siRNA, TAK1 siRNA, or CaMKKβ siRNA, and they were treated with 2 μM Pep A for 24 h. **H** A549 and Caki cells were treated with the indicated concentrations of Pep A for 24 h. **I** Caki cells were pretreated with the indicated concentrations of SP600125 for 30 min, and they were then treated with 2 μM Pep A for 24 h. Protein expression was measured via western blotting (**A**–**I**).

Next, we examined how Cat D inhibition increases autophagic flux. ULK1 kinase is an initiating autophagy-related kinase, and the regulation of ULK1 phosphorylation plays a central role in autophagy. AMPK increases the phosphorylation of S317/S777, which is essential for ULK1 activation, and mTOR prevents ULK1 activation via phosphorylation of S757 [33]. Therefore, we investigated whether inhibition of Cat D modulates ULK1 activation. Cat D KO/ KD decreased the phosphorylation of ULK1 at S757 and increased the phosphorylation of ULK1 at S777 in all tested cancer cell lines (Fig. 5A). In line with these results, Cat D KO/KD inhibited mTOR phosphorylation and induced AMPK phosphorylation (Fig. 5B). To confirm the involvement of mTOR dephosphorylation in Pep A-induced NF-κB activation, we used the mTOR specific inhibitor (everolimus). Everolimus more increased p65 phosphorylation and IκB degradation (Fig. 5C). As AMPK suppresses mTOR signaling, we examined the effect of AMPK signaling inhibition on autophagy. AMPK KD decreased LC3 II formation via ULK1 inhibition and mTOR dephosphorylation in Pep A-treated cells (Fig. 5D). Further, the AMPK inhibitor Compound C and AMPK KD also blocked LC3 II formation and IκB degradation (Fig. 5E, F). These results indicated that Cat D inhibition increases autophagy flux via AMPK-dependent modulation of ULK1 phosphorylation.

Three upstream kinases LKB1, CaMKKβ, and TAK1 modulate AMPK activation [34, 35]. Therefore, we investigated the kinases that are involved in Cat D inhibition-mediated IκB degradation through modulation of autophagy flux. As shown in Fig. 5G, LKB1 KD only blocked the degradation of IκB by inhibiting dephosphorylation of ULK1 at S757 and LC3 lipidation in Pep A-treated cells. Neither TAK1 nor CaMKKβ altered IκB degradation following Pep A treatment. Additionally, the LKB1-deficient cell line A549 was resistant to Pep A-treated IκB degradation (Fig. 5H). Furthermore, the JNK inhibitor did not affect AMPK phosphorylation (Fig. 5I), and AMPK KD did not alter the levels of JNK phosphorylation (Fig. 5D), indicating that the two pathways are independent. In addition, we also confirmed that Bcl-xL inhibitor (Navitoclax) did not change phosphorylation of p65 and IκB degradation (Supplementary Fig. S1). Our data suggested that the LKB1-AMPK-ULK1 signaling pathway plays a critical role in IκB degradation through Cat D inhibition via the modulation of autophagy.

### Autophagy-dependent degradation of IκB increases RNF183 E3 ligase expression

**Fig. 6:**
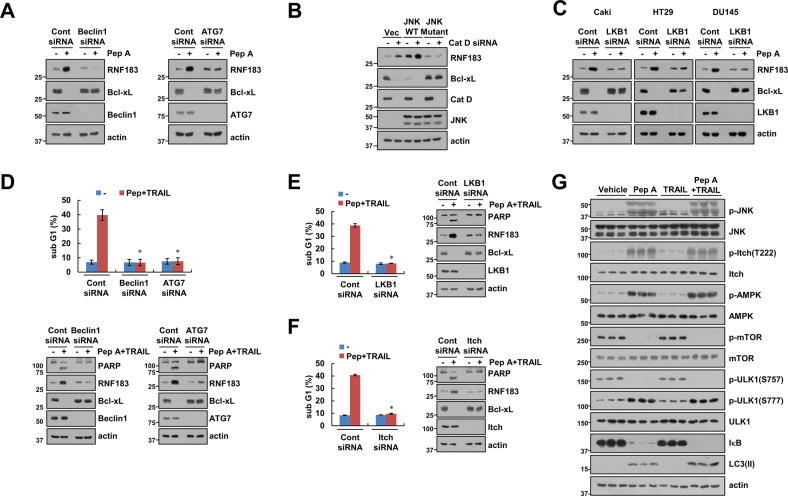
Autophagy-dependent degradation of IκB increases RNF183 expression. **A** Caki cells were transiently transfected with Cont siRNA, Beclin1 siRNA, and ATG7 siRNA; they were further treated with 2 μM Pep A for 24 h. **B** Caki cells were transiently co-transfected with Cont siRNA or Cat D siRNA and JNK WT or JNK mutant for 24 h. **C** Cancer cells (Caki, HT29, and DU145) cells were transiently transfected with Cont siRNA or LKB1 siRNA, and they were then treated with 2 μM Pep A for 24 h. **D**–**F** Caki cells were transiently transfected with Cont siRNA, Beclin1 siRNA (**D**), ATG7 siRNA (**D**), LKB1 siRNA (**E**), and Itch siRNA (**F**), and they were further treated with 2 μM Pep A and 50 ng/mL TRAIL for 24 h. **G** Mice were treated with 5 mg/kg Pep A, 3 mg/kg GST-TRAIL, a combination of Pep A and GST-TRAIL, or vehicle for 16 days. Protein expression was measured via western blotting (**A**–**G**). Apoptosis was measured via flow cytometry (**D**–**F**). Values in the graphs (**D**–**F**) represent mean ± SD of three independent experiments. *p < 0.01 compared to Pep A plus TRAIL in Cont siRNA.

Previously, we reported that inhibition of Cat D sensitized cancer cells to anticancer drugs through RNF183-mediated Bcl-xL degradation [16], and activation of NF-κB signaling is important for the induction of RNF183 expression. Therefore, we investigated whether the upstream signaling of IκB degradation is involved in the modulation of RNF183 expression. KD of autophagy-related genes *Beclin1* and *ATG7* significantly blocked Pep A-induced RNF183 upregulation and Bcl-xL downregulation (Fig. 6A). Additionally, the catalytically inactive mutant JNK and LKB1 KD blocked Pep A-mediated RNF183 upregulation and Bcl-xL downregulation (Fig. 6B, C). Next, we confirmed whether the regulatory mechanism of RNF183 expression controls sensitivity to anticancer drugs. KD of the autophagy-related genes *Beclin1* and *ATG7*, LKB1, and Itch significantly inhibited the combined treatment of Pep A plus TRAIL-induced apoptosis, PARP cleavage, and Bcl-xL downregulation (Fig. 6D–F). Next, we detected similar results using lysates from the in vivo xenograft models (Fig. 6G). Therefore, our results suggested that Cat D inhibition activates the NF-κB signaling pathway, which is related to anticancer activity, via autophagy-dependent IκB degradation.

### Cat D indicates inverse correlation with levels of phospho-Itch and LKB1 in human renal clear carcinoma (RCC) tissues

**Fig. 7:**
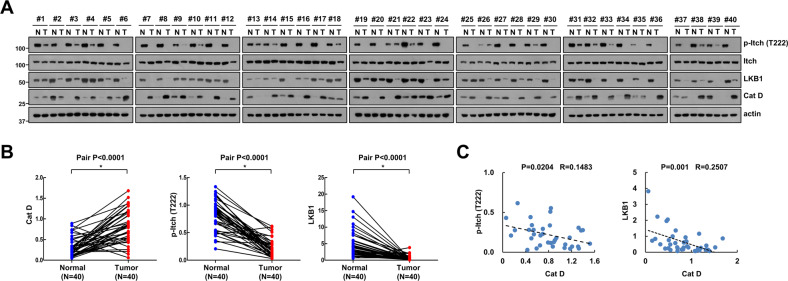
The inverse correlation between Cat D and phospho-Itch or LKB1 expression in renal tumor tissues. **A**, **B** Analysis of protein expression in 40 paired primary renal clear carcinoma tissues as compared with the adjacent normal tissues. **C** The inverse correlation between Cat D and phospho-Itch or LKB1 was observed at the protein levels.

We investigated protein level of phospho-Itch and LKB1 in human renal clear carcinoma (RCC) tissues. When Cat D (82.5%, 33/40) is highly expressed in RCC tumor tissues, phospho-Itch (92.5%, 37/40) and LKB1 (85%, 34/40) were downregulated (Fig. 7A, B). In addition, we found that phospho-Itch and LKB1 has inverse correlationship with levels of Cat D expression in RCC (Fig. 7C).

## Discussion

**Fig. 8:**
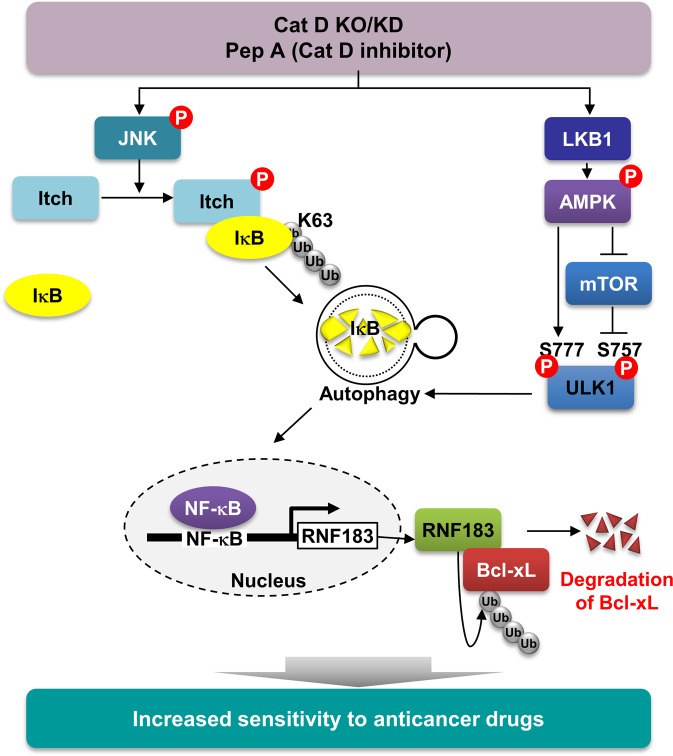
Scheme indicating the mechanism of Cat D inhibition-induced sensitizing effect on anticancer drugs via activation of NF-κB signaling. Two molecular mechanisms are associated with autophagy-mediated IκB degradation by Cat D inhibition. First, depletion of Cat D phosphorylates Itch at T222 through activation of JNK signaling. Second, LKB1-mediated AMPK activation by Cat D inhibition phosphorylates ULK1 at S777, while ULK1 at S757 is dephosphorylated by inhibition of mTOR signaling. Subsequently, autophagy-dependent IκB degradation is involved in Cat D inhibition-mediated sensitizing effect to anticancer drugs through RNF183-dependent Bcl-xL downregulation.

In this study, we demonstrated the molecular mechanism of NF-κB activation via Cat D inhibition. Inhibition of Cat D significantly increased IκB degradation in an autophagy-lysosome-dependent manner. Further, inhibition of Cat D increased K63-linked ubiquitination of IκB by Itch E3 ligase. LKB1-AMPK-ULK1 signal axis-mediated autophagy activation is involved in IκB degradation. Autophagy-dependent IκB degradation-mediated NF-κB activation increased RNF183 expression, which was associated with Cat D inhibition-induced Bcl-xL downregulation (Fig. 8).

Itch is a HECT-type E3 ligase containing a C2 domain, four WW motifs, and a HECT domain [36]. Itch was present in the central region with WW whereas PRR combined with C-terminal HECT and its activity was suppressed. When phosphorylated to the PRR motif under certain conditions, the HECT domain was exposed, since the intracellular interaction weakens through conformational changes. It is known that the catalytic activity of HECT domain increases significantly [28]. Phosphorylation of Itch is regulated by JNK. JNK phosphorylates S199, T222, and S232 residues in the PRR motif to regulate Itch activity. In our study, inhibition of Cat D significantly increased JNK phosphorylation (Fig. 3B), and a JNK inhibitor SP600125 completely blocked Itch T222 phosphorylation (Fig. 3C). Inhibition of Itch phosphorylation also reduced the binding of IκB to Itch (Fig. 3E). Although inhibition of Cat D markedly increased JNK phosphorylation, the upstream kinase of JNK in our system was unclear. Reactive oxygen species (ROS) are candidates for activation of JNK signaling. However, since Cat D inhibition did not increase ROS production [16], we ruled out the possibility of ROS being upstream of JNK. Additionally, we found that AMPK KD did not alter JNK phosphorylation by inhibiting Cat D (Fig. 5D). Further investigations are required to clearly identify the upstream kinases involved in Cat D inhibition-mediated JNK activation.

ULK1 activation is associated with autophagy initiation in mammalian cells. ULK1 forms a complex with autophagy-related protein 13 (ATG13), focal adhesion kinase family interacting protein of 200 kDa (FIP200), and ATG101, and its kinase activity is essential for autophagy initiation through phosphorylation of ULK1, ATG13, FIP200, and ATG101 [37–40]. ULK1 activation is modulated by phosphorylation, and mTOR and AMPK mainly phosphorylate ULK1. Phosphorylation regulation by the two kinases plays an important role in determining the protein bound to ULK1. For example, phosphorylation of ULK1 at S757 prevents AMPK interaction, and AMPK-mediated mTOR inactivation decreases the phosphorylation of ULK1 at S757, resulting in interaction with AMPK and phosphorylation of ULK1 at S317 and S777 [33]. In our study, inhibition of Cat D increased the phosphorylation of ULK1 at S777 whereas phosphorylation at S757 was suppressed (Fig. 5A). It was confirmed that phosphorylation of ULK1 due to Cat D inhibition was blocked by AMPK KD, and dephosphorylation of mTOR was also inhibited by AMPK KD (Fig. 5D). Therefore, AMPK activation via Cat D inhibition plays a key role in autophagy induction. We also identified an upstream kinase of AMPK. There are three known upstream kinases of AMPK, LKB1, TAK1, and CaMKKβ. Among them, it is known that LKB1 acts as an upstream kinase of AMPK as the phenomena that occurred via Cat D inhibition disappeared only when LKB1 was knocked out (Fig. 5G). LKB1 is a serine/threonine kinase known to be a tumor suppressor gene. Mutations in LKB1 have been found in sporadic cancers and Peutz-Jeghers syndrome [41]. By contrast, LKB1 has oncogenic functions. For example, LKB1-deficient cells promote NADPH depletion and increase H_2_O_2_ production, resulting in the induction of cell death [42], and the loss of LKB1 makes the cells resistant to oncogene-induced transformation [43]. Our findings also confirmed that sensitivity to anticancer drugs increased in the absence of LKB1 via downregulation of Bcl-xL, indicating that at least the activity of LKB1 must be suppressed to increase cancer cell death (Fig. 6E). LKB1 activation is regulated by the formation of a heterotrimeric complex with STE20-related kinase adaptor (STRAD) and mouse protein 25 (MO25), both of which maintain the activated conformation of LKB1 [44]. One of the mechanisms known to regulate the activity of LKB1 is ubiquitination by Skp2-SCF ligase. K63-linked ubiquitination of LKB1 increased the integrity of LKB1-STRAD-MO25 complex [45], and SUMOylation of LKB1 at K178 also regulates its activity via modulation of LKB1-AMPK interaction [46] and localization [47]. LKB1 KD dramatically inhibited Pep A-induced RNF183 upregulation and Bcl-xL downregulation (Fig. 6C). Furthermore, LKB1 KD significantly inhibited the combined treatment of Pep A and TRAIL-induced apoptosis (Fig. 6E). Therefore, the LKB1-AMPK-ULK1 axis is a key signaling pathway in Cat D inhibition-induced RNF183 upregulation. However, further studies are needed to determine how Cat D inhibition regulates the activity of LKB1.

In previous study, we reported that inhibition of Cat D sensitizes cancer cells to anticancer drugs via RNF183-mediated downregulation of Bcl-xL expression [16]. Here, we identified the mechanism of RNF183 upregulation by Cat D inhibition, and phosphorylation of Itch and activation of LKB1 have critical roles in upregulation of RNF183. We also found that Cat D had positive correlation with Bcl-xL expression and had inverse correlation with RNF183, p-Itch at T222 and LKB1 (Fig. 7) [16]. Since the expression level of Cat D is correlated with the expression of anti-apoptotic Bcl-xL, it means that it can exhibit resistance to anticancer drugs. Chemotherapy resistance of neuroblastomas with amplified MYCN was related with Cat D expression, and they suggest that enhancement of Bcl-2 anti-apoptotic function [11]. In addition, Cat D deficiency in the mammary epithelium delayed breast cancer progression and induced quiescent state by inhibiting mTORC1 signaling [15]. Inhibition of Cat D markedly increased apoptosis by sub-lethal dosage of sorafenib and sunitinib or paclitaxel in Caki and prostate carcinoma DU145 cells (Supplementary Fig. S2). Sorafenib and sunitinib are approved for renal or prostate cell cancer treatment, respectively. Therefore, therapeutic strategies targeting Cat D may increase the sensitivity of anticancer drugs.

In conclusion, we clearly showed that Cat D inhibition activated NF-κB signaling via IκB degradation by induction of K63-linked ubiquitination of IκB using Itch E3 ligase and autophagy flux using LKB1-AMPK-ULK1 activation. Based on our findings, we suggest that inhibition of Cat D can be a target mechanism for controlling sensitivity to anticancer drugs through these novel mechanisms.

## Supplementary information

supplementary information

checklist

## Data Availability

The datasets used and analyzed in this study are available from the corresponding author.
