# Supplementary material for: Itch and autophagy-mediated NF-κB activation contributes to inhibition of cathepsin D-induced sensitizing effect on anticancer drugs
Source: Cell Death Dis. 2022 Jun 17;13(6):552. doi: 10.1038/s41419-022-05011-4 (PMC9205942; doi:10.1038/s41419-022-05011-4)
Supplement: Supplementary file 1 — supplementary information [file 41419_2022_5011_MOESM1_ESM.docx]

**Supplemental material**

**Supplemental Figure S1**

**Supplemental Figure S2**

**Supplemental Table S1**

**
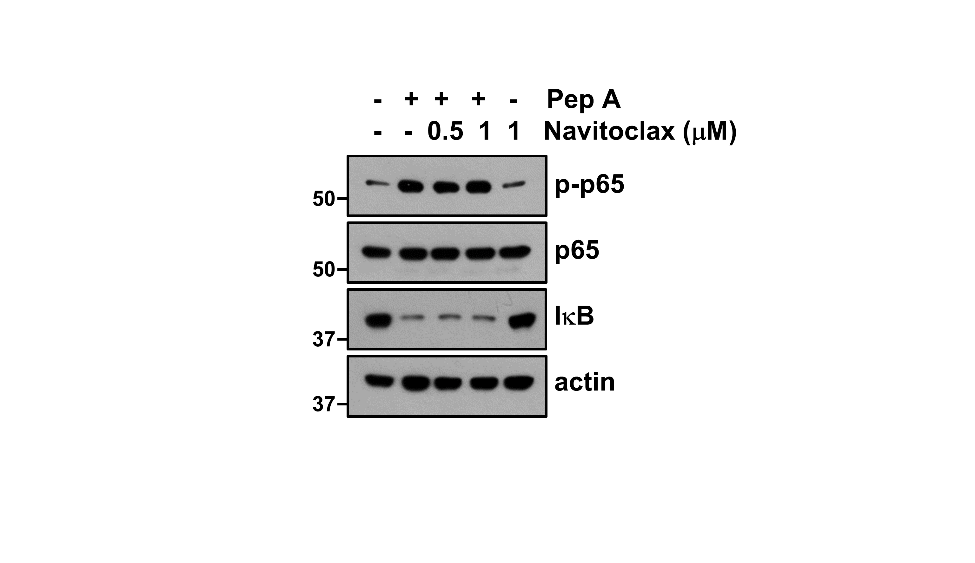
**

**Fig. S1** **Inhibitor of Bcl-xL has no effect on pep A-induced activation of NF-κB.** Caki cells were pretreated with the indicated concentrations of Navitoclax, and 2 μM Pep A was then added for 24 h. Protein expression was measured via western blotting.

**
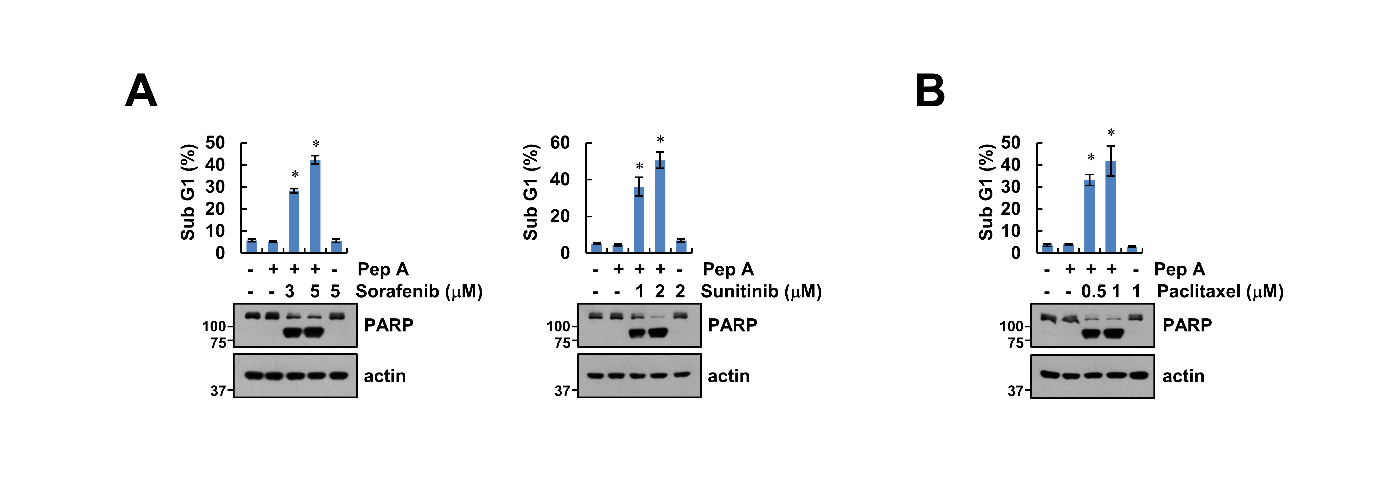
**

**Fig. S2 Inhibition of Cat D sensitizes cancer cells to anticancer drugs. A** Renal carcinoma Caki cells (**A**) and prostate carcinoma DU145 cells (**B**) were treated with the indicated concentrations of Sorafenib, Sunitinib, or Pacilitaxel in the presence or absence of 2 μM Pep A for 24 h. Protein expression was measured via western blotting (**A, B**). Apoptosis was measured via flow cytometry (**A, B**). Values in the graphs (**D-F**) represent mean ± SD of three independent experiments. **p* < 0.01 compared to the control.

**Supplementary Table S1. Reagent and Antibody**

| **Reagent or Antibody** | **Source** | **Identifier** |
| --- | --- | --- |
| Bafilomycin A1 | Sigma-Aldrich | Cat#B1793 |
| Chloroquine | Sigma-Aldrich | Cat#415480 |
| Clomipramine | Sigma-Aldrich | Cat#C7291 |
| Cisplatin | Sigma-Aldrich | Cat#P4394 |
| Compound C | Calbiochem | Cat#171261 |
| Cycloheximide (CHX) | Sigma-Aldrich | Cat#01810 |
| Everolimus | Sigma-Aldrich | Cat#SML2282 |
| Lactacystin | Enzo Life Sciences | Cat#BML-PI104 |
| MG132 | Sigma-Aldrich | Cat#M8699 |
| Navitoclax | Selleckchem | Cat#S1001 |
| Paclitaxel | Sigma-Aldrich | Cat#T7402 |
| Pepstatin A | Enzo Life Sciences | Cat#ALX-260-085 |
| Sorafenib | Santa Cruz Biotechnology | Cat#SC-357801A |
| SP600125 | Enzo Life Sciences | Cat#BML-EI305 |
| Sunitinib | Tocris Bioscience | Cat#3768 |
| TNF-α | R&D Systems | Cat#P01375 |
| Torin | Tocris Bioscience | Cat#4247 |
| TRAIL | R&D Systems | Cat#375-TL |
| AMPK | Santa Cruz Biotechnology | Cat#sc-25792 |
| ATG7 | ProSci Incoporated | Cat#3617 |
| Bcl-xL | Cell Signaling Technology | Cat# 2764 |
| Beclin-1 | Santa Cruz Biotechnology | Cat#11427 |
| CaMKKβ | Santa Cruz Biotechnology | Cat#sc-271674 |
| Cathepsin D | Santa Cruz Biotechnology | Cat#sc-6486 |
| Itch | Santa Cruz Biotechnology | Cat#28367 |
| IκB | Cell Signaling Technology | Cat#9242 |
| JNK | Cell Signaling Technology | Cat#9252 |
| K63-Ub | Cell Signaling Technology | Cat#12930 |
| LC3 | MBL | Cat#PD014 |
| LKB1 | Cell Signaling Technology | Cat#3047 |
| mTOR | Cell Signaling Technology | Cat#2972 |
| NF-κB p65 | Santa Cruz Biotechnology | Cat#sc-8008 |
| p-AMPK(thr172) | Cell Signaling Technology | Cat#8208 |
| p-Itch (thr222) | EMD Millipore | Cat#AB10050 |
| p-JNK | Cell Signaling Technology | Cat#9251 |
| p-mTOR (ser2448) | Cell Signaling Technology | Cat#5536 |
| p-p65 (ser276) | Cell Signaling Technology | Cat#3037 |
| p-ULK1 (ser757) | Cell Signaling Technology | Cat#14202 |
| p-ULK1 (ser777) | EMD Millipore | Cat#ABC213 |
| PARP | Cell Signaling Technology | Cat#9542 |
| RNF183 | Invitrogen | Cat#PA5-23862 |
| TAK1 | Santa Cruz Biotechnology | Cat#sc-7162 |
| TFEB | Abcam | Cat#ab220695 |
| Ub | Santa Cruz Biotechnology | Cat#sc-8017 |
| Ub-HRP | Enzo Life Sciences | Cat#BML-PW0150-0100 |
| ULK1 | Cell Signaling Technology | Cat#6439 |
| β-actin | Sigma-Aldrich | Cat#A2228 |
| β-TrCP | Santa Cruz Biotechnology | Cat#1712 |
| **siRNAs and shRNA** |  |  |
| AMPK siRNA | Santa Cruz Biotechnology | Cat#sc-29673 |
| ATG7 siRNA | Santa Cruz Biotechnology | Cat#sc-41447 |
| Beclin-1 siRNA | Santa Cruz Biotechnology | Cat#sc-29797 |
| CaMKKβ siRNA | Santa Cruz Biotechnology | Cat#sc-38955 |
| Cathepsin D siRNA | Santa Cruz Biotechnology | Cat#sc-29239 |
| GFP (control) siRNA | Bioneer | 5`- GUU CAG CGU GUC CGG CGA G -3` |
| Itch siRNA | Santa Cruz Biotechnology | Cat#sc-40364 |
| LKB1 siRNA | Santa Cruz Biotechnology | Cat#sc-35816 |
| β-TrCP siRNA | Santa Cruz Biotechnology | Cat#sc-37178 |
| Non-target shRNA | Thermo Fisher Scientific |  |
| Itch shRNA | Thermo Fisher Scientific |  |
| **Plasmids** |  |  |
| Itch/C832G | Addgene | Cat#11428 |
| Itch WT | Addgene | Cat#11427 |
| mRFP-EGFP-LC3 | Addgene | Cat#117413 |
| pRK5-HA-Ubiquitin | Addgene | Cat# 17608 |
